# Supplementary material for: Interprofessional Educational Interventions to Improve Pharmacological Knowledge and Prescribing Competency in Medical Students and Trainees: A Scoping Review
Source: Pharmacy (Basel). 2025 Aug 27;13(5):116. doi: 10.3390/pharmacy13050116 (PMC12452605; doi:10.3390/pharmacy13050116)
Supplement: Supplementary file 1 [file pharmacy-13-00116-s001.zip › Supplementary Materials S1- Search Strategies.pdf]

Supplementary Materials S1

Ovid MEDLINE(R) ALL <1946 to February 14, 2025>

1 exp Interprofessional Education/ or exp Interprofessional Relations/ or (interprofessional education or interdisciplinary education or multidisciplinary education or professional collaboration or team-based learning or collaborative learning or interprofessional teamwork or interprofessional teaching or interprofessional training or interprofessional learning or IPE).tw,kf. 80670

2 Clinical Competence/ or Competency-Based Education/ or Educational Measurement/ or (work based assessment* workplace-based assessment* or WBA* or clinical evaluation exercise or CEX* or procedure based assessment* or PBA* or Entrustable professional activit* or Entrustability or EPA* or entrustment scales or authentic assessment or formative assessment* tool*).tw,kf. 188307

3 (interprofessional education or interprofessional collaboration or interprofessional teaching or interprofessional training or interprofessional learning or interprofessional relation* or IPE or professional education or professional collaboration* or interdisciplinary education or educational intervention* or collaborative education).tw,kf. 33044

4 1 or 2 or 3 284506

5 exp Students, Medical/ or exp schools, medical/ or exp education, medical/ 229789

6 ((medicine or medical) adj3 (student* or school*)).tw,kf. 110351

7 (intern or interns or internship* or resident* or residency or junior doctor* or medical graduate* or graduate doctor* or specialist trainee* or medical trainee* or registrar*).tw,kf. 304229

8 5 or 6 or 7 521604

9 exp pharmacology/ or exp prescriptions/ or exp education, pharmacy/ 162827

10 (prescription* or prescrib* or pharmacy or pharmacolog* or pharmaceut* or pharmacotherap* or pharmacist* or medication* or drug*).tw,kf. 3200645

11 9 or 103242869

12 4 and 8 and 11 3440

13 limit 12 to english language 3279

14	limit 13 to yr="2020 -Current"	729

Embase Classic+Embase <1947 to 2025 February 14>

1 exp Interprofessional Education/ or exp Interprofessional Relations/ or (interprofessional education or interdisciplinary education or multidisciplinary education or professional collaboration or team-based learning or collaborative learning or interprofessional teamwork or interprofessional teaching or interprofessional training or interprofessional learning or IPE).tw,kf. 76017

2 clinical competence/ or (work based assessment* workplace-based assessment* or WBA* or clinical evaluation exercise or CEX* or procedure based assessment* or PBA* or Entrustable professional activit* or Entrustability or EPA* or entrustment scales or authentic assessment or formative assessment* tool*).tw,kf. 127105

3 (interprofessional education or interprofessional collaboration or interprofessional teaching or interprofessional training or interprofessional learning or interprofessional relation* or IPE or professional education or professional collaboration* or interdisciplinary education or educational intervention* or collaborative education).tw,kf. 43063

4 1 or 2 or 3 230659

5 exp Students, Medical/ or exp schools, medical/ or exp education, medical/ 471396

6 ((medicine or medical) adj3 (student* or school*)).tw,kf. 152410

7 (intern or interns or internship* or resident* or residency or junior doctor* or medical graduate* or graduate doctor* or specialist trainee* or medical trainee* or registrar*).tw,kf. 404881

8 5 or 6 or 7 841695

9 exp pharmacology/ or exp prescriptions/ or exp education, pharmacy/ 5949454

10 (prescription* or prescrib* or pharmacy or pharmacolog* or pharmaceut* or pharmacotherap* or pharmacist* or medication* or drug*).tw,kf. 4652578

11 9 or 108680943

12 4 and 8 and 11 4159

13 limit 12 to english language 4035

14 limit 13 to yr="2020 -Current" 1230

EBM Reviews - Cochrane Central Register of Controlled Trials <December 2024>

1 exp professional education/ or exp Interprofessional Education/ or exp interprofessional relations/ or (interprofessional education or interdisciplinary education or multidisciplinary education or professional collaboration or team-based learning or collaborative learning or interprofessional teamwork or interprofessional teaching or interprofessional training or interprofessional learning or IPE).tw,kf. [mp=title, original title, abstract, floating sub-heading word, mesh headings, heading words, keyword] 8806

2 exp Clinical Competence/ or exp Educational Measurement/ or (work based assessment* workplace-based assessment* or WBA* or clinical evaluation exercise or CEX* or procedure based assessment* or PBA* or Entrustable professional activit* or Entrustability or EPA* or entrustment scales or authentic assessment or formative assessment* tool*).tw,kf. 12128

3 (interprofessional education or interprofessional collaboration or interprofessional teaching or interprofessional training or interprofessional learning or interprofessional relation* or IPE or professional education or professional collaboration* or interdisciplinary education or educational intervention* or collaborative education).tw,kf. 7042

4 1 or 2 or 3 23204

5 exp Students, Medical/ or exp schools, medical/ or exp education, medical/ 5597

6 ((medicine or medical) adj3 (student* or school*)).tw,kf. 7837

7 (intern or interns or internship* or resident* or residency or junior doctor* or medical graduate* or graduate doctor* or specialist trainee* or medical trainee* or registrar*).tw,kf. 19751

8 5 or 6 or 7 28237

9 exp pharmacology/ or exp prescriptions/ or exp education, pharmacy/ 4265

10 (prescription* or prescrib* or pharmacy or pharmacolog* or pharmaceut* or pharmacotherap* or pharmacist* or medication* or drug*).tw,kf. 434973

11 9 or 10436649

12 4 and 8 and 11 570

13 limit 12 to english language 565

14	limit 13 to yr="2020 -Current"	81

APA PsycInfo <1806 to February 2025 Week 1>

1 exp collaborative learning/ or ("interprofessional education" or "interprofessional relation*" or "interprofessional collaboration" or "interprofessional teaching" or "interprofessional training" or "interprofessional learning" or "interprofessional teamwork" or "interdisciplinary education" or "team-based learning" or "collaborative learning" or "collaborative education" or "professional collaboration*" or "professional education" or "educational intervention*" or IPE).ti,ab,de. 20584

2 educational assessment/ or professional competence/ or personnel evaluation/ or ("work based assessment*" or "workplace-based assessment*" or WBA* or "clinical evaluation exercise" or CEX* or "procedure based assessment*" or PBA* or "entrustable professional activit*" or entrustability or EPA* or "entrustment scales" or "authentic assessment" or "formative assessment* tool*").ti,ab,de. 18135

3 1 or 2 38303

4 exp Medical Students/ or exp Medical Education/ or ((medicine or medical) adj3 (student* or school*)).ti,ab,de. [mp=title, abstract, heading word, table of contents, key concepts, original title, tests & measures, mesh word] 44910

5 (intern or interns or internship* or resident* or residency or "junior doctor*" or "medical graduate*" or "graduate doctor*" or "specialist trainee*" or "medical trainee*" or registrar*).ti,ab,de. 97528

6 4 or 5 131931

7 exp Pharmacy/ or exp "Prescribing (Drugs)"/ or exp pharmacology/ or (prescription* or prescrib* or pharmacy or pharmacolog* or pharmaceut* or pharmacotherap* or pharmacist* or medication* or drug*).ti,ab,de. 524623

8 3 and 6 and 7 404

9 limit 8 to english language 399

10 limit 9 to yr="2020 -Current" 103

Scopus

(TITLE-ABS-KEY ("interprofessional education") OR TITLE-ABS-KEY ("interprofessional collaboration") OR TITLE-ABS-KEY ("interdisciplinary education") OR TITLE-ABS-KEY ("professional collaboration") OR TITLE-ABS-KEY ("team-based learning") OR TITLE-ABS-KEY ("collaborative learning") OR TITLE-ABS-KEY ("interprofessional teamwork") OR TITLE-ABS-KEY ("interprofessional teaching") OR TITLE-ABS-KEY ("interprofessional training") OR TITLE-ABS-KEY ("interprofessional learning") OR TITLE-ABS-KEY ("IPE"))

AND

(TITLE-ABS-KEY ("medical student*") OR TITLE-ABS-KEY ("medicine student*") OR TITLE-ABS-KEY ("medical trainee*") OR TITLE-ABS-KEY ("medical intern*") OR TITLE-ABS-KEY ("resident doctor*") OR TITLE-ABS-KEY ("junior doctor*") OR TITLE-ABS-KEY ("graduate doctor*") OR TITLE-ABS-KEY ("specialist trainee*") OR TITLE-ABS-KEY ("medical registrar*") OR TITLE-ABS-KEY ("pharmacy student*") OR TITLE-ABS-KEY ("nursing student*"))

AND

(TITLE-ABS-KEY ("pharmacology education") OR TITLE-ABS-KEY ("prescribing education") OR TITLE-ABS-KEY ("prescription training") OR TITLE-ABS-KEY ("pharmacology curriculum") OR TITLE-ABS-KEY ("medication safety") OR TITLE-ABS-KEY ("prescribing competency") OR TITLE-ABS-KEY ("pharmacist training") OR TITLE-ABS-KEY ("pharmaceutical education") OR TITLE-ABS-KEY ("drug education") OR TITLE-ABS-KEY ("medication error*") OR TITLE-ABS-KEY ("prescribing error*") OR TITLE-ABS-KEY ("work based assessment*") OR TITLE-ABS-KEY ("workplace-based assessment*") OR TITLE-ABS-KEY ("WBA*") OR TITLE-ABS-KEY ("clinical evaluation exercise") OR TITLE-ABS-KEY ("CEX*") OR TITLE-ABS-KEY ("procedure based assessment*") OR TITLE-ABS-KEY ("PBA*") OR TITLE-ABS-KEY ("entrustable professional activit*") OR TITLE-ABS-KEY ("entrustability") OR TITLE-ABS-KEY ("EPA*") OR TITLE-ABS-KEY ("entrustment scales") OR TITLE-ABS-KEY ("authentic assessment") OR TITLE-ABS-KEY ("formative assessment* tool*"))

AND

PUBYEAR > 2019 AND PUBYEAR < 2026

AND

(LIMIT-TO (LANGUAGE , "English"))

CINAHL

S1 (MH "Education, Interdisciplinary") OR (MH "Interprofessional Relations") OR ("interprofessional education" OR "interprofessional collaboration" OR "interdisciplinary education" OR "professional collaboration" OR "team-based learning" OR "collaborative learning" OR "interprofessional teamwork" OR "interprofessional teaching" OR "interprofessional training" OR "interprofessional learning" OR "IPE"

S2 (MH "Competency Assessment") OR (MH "Student Performance Appraisal+") OR ("work-based assessment*" OR "WBA" OR "clinical evaluation exercise" OR "CEX" OR "procedure-based assessment*" OR "PBA" OR "Entrustable Professional Activit*" OR "Entrustability"

S3 S1 OR S2

S4 (MH "Students, Medical") OR (MH "Education, Medical") OR (MH "Schools, Medical") OR ((medicine OR medical) N3 (student* OR trainee* OR school*)) OR ("intern" OR "interns" OR "internship*" OR "resident*" OR "residency" OR "junior doctor*" OR "medical graduate*" OR "graduate doctor*" OR "specialist trainee*" OR "registrar*")

S5 (MH "Pharmacy and Pharmacology") OR (MH "Education, Pharmacy") OR (MH "Prescriptions, Drug+") OR (MH "Inappropriate Prescribing") OR ("prescription*" OR "prescrib*" OR "pharmacy" OR "pharmacolog*" OR "pharmaceut*" OR "pharmacotherap*" OR "pharmacist*" OR "medication*" OR "drug*")

S6 s3 AND s4 AND s5

ERIC

S1 ((DE "Cooperative Planning") OR (DE "Interdisciplinary Approach")) OR (DE "Interpersonal Relationship")) OR ("interprofessional education" OR "interprofessional collaboration" OR "interdisciplinary education" OR "professional collaboration" OR "team-based learning" OR "collaborative learning" OR "interprofessional teamwork" OR "interprofessional teaching" OR "interprofessional training" OR "interprofessional learning" OR "IPE" OR "multidisciplinary education"

S2 ((DE "Performance Based Assessment") OR (DE "Competency Based Education") OR ("work-based assessment*" OR "WBA" OR "clinical evaluation exercise" OR "CEX" OR "procedure-based assessment*" OR "PBA" OR "Entrustable Professional Activit*" OR "Entrustability" ((DE "Performance Based Assessment") OR (DE "Competency Based Education") OR ("work-based assessment*" OR "WBA" OR "clinical evaluation exercise" OR "CEX" OR "procedure-based assessment*" OR "PBA" OR "Entrustable Professional Activit*" OR "Entrustability"

S3 S1 or S2

S4 (DE "Medical Education" OR DE "Graduate Medical Education" OR DE "Clinical Teaching (Health Professions)" OR DE "Medical Schools" OR DE "Medical Students") ((medicine OR medical) N3 (student* OR trainee* OR school*)) OR ("intern" OR "interns" OR "internship*" OR "resident*" OR "residency" OR "junior doctor*" OR "medical graduate*" OR "graduate doctor*" OR "specialist trainee*" OR "registrar*")

S5 DE "Pharmacy" OR DE "Medicine" OR DE "Pharmaceutical Education" OR DE "Pharmacology" OR ("prescription*" OR "prescrib*" OR "pharmacy" OR "pharmacolog*" OR "pharmaceut*" OR "pharmacotherap*" OR "pharmacist*" OR "medication*" OR "drug*")

S6 S3 and S4 and S5
