# Supplementary material for: Interprofessional Educational Interventions to Improve Pharmacological Knowledge and Prescribing Competency in Medical Students and Trainees: A Scoping Review
Source: Pharmacy (Basel). 2025 Aug 27;13(5):116. doi: 10.3390/pharmacy13050116 (PMC12452605; doi:10.3390/pharmacy13050116)
Supplement: Supplementary file 1 [file pharmacy-13-00116-s001.zip › Supplmentary Materials Table S2-Data Extraction.pdf]

Table S2: Summary of included studies

Study	Country	Study Design	Participants (n=)	Intervention	Focus Area	Outcome measures	Assessment timepoints	Kirkpatrick/Barr Level	Results
**Case-based learning**									
**[31]**	USA	Pre-post intervention	200 second-year medical students (prescription writing plenary session)<br><br>36 third-year medical students (nonprescription case-based sessions)<br><br>22 fourth year-pharmacy students as teachers	Two interprofessional activities: (1) Pharmacy students taught prescription writing in a 3.5-hour workshop for second-year medical students. The first hour involved a one-hour lecture on prescription writing by pharmacy faculty member. In the workshop, 2 pharmacy students would facilitate 12-15 medical student groups through prescription exercises: (2) Two-session 2-hour case-based nonprescription product teaching module led by pharmacy students for third-year medical students.	Prescription writing and nonprescription medication counselling (Prescribing skills)	Objective: Prescription-writing OSCE (pass/fail)<br>No formal test for nonprescription medicine module<br><br>Subjective: Pre/post confidence surveys 5-point Likert scale	Immediately before and after	Level 1<br><br>Level 2a<br><br>Level 2b<br><br>Level 2b	90% of medical students found the workshop to be 'helpful'" or 'extremely helpful'<br><br>Medical students recognised pharmacists as playing an important role in the healthcare team before (93%) and after (98%)<br><br>Significant improvement in medical students' self-reported confidence in prescription writing (e.g., 6% → 64% for prescriptions containing all legally required elements, p<.001); all passed related OSCE at the end of semester.<br><br>Third-year students in the OTC selective reported significant gains in familiarity with OTC resources (47% → 96%, p<.001), confidence in making OTC recommendations (52% → 96%, p<.001), and counselling (52% → 80%, p<.001).

**[47]**	Hong Kong	Post-intervention	117 fourth-year medical students, 108 third-year nursing students and 30 fourth-year pharmacy students.	Interprofessional teams composed of 5-7 students, with at least 2 medical students, two nursing students and one pharmacy student participated in a 3.5-hour face-to-face session on IPE anticoagulation therapy.	Anticoagulation therapy and interprofessional collaboration (interprofessional collaboration)	Subjective: Various scales to assess factors relating to autonomy, competence and relatedness, team effectiveness, collective dedication, behavioural engagement and goal achievements	Immediately after	Level 2a	A student's sense of autonomy significantly predicted IPE outcomes: team effectiveness, collective dedication, behavioural engagement and goal achievement.
**[14]**	USA	Pre-post intervention	312 health professions students: 60 medicine (20%) 124 pharmacy (41%)<br>92 nursing (31%)<br>34 social work (11%)<br>2 addiction studies (0.7%)	Two-hour interprofessional education session on opioid use and chronic pain. Students worked in interprofessional groups on an unfolding patient case, including interpretation of clinical data, videos, and collaborative treatment planning. Developed by an interprofessional faculty team.	Opioid use and pain management; interprofessional collaboration (interprofessional collaboration)	Objective: 10-item opioid knowledge pre-test<br><br>Subjective: 17 item survey, 8 items related to IP attitudes, 8 on related to confidence and attitudes to opioid knowledge, and two open ended questions	Immediately before and after	Level 1<br><br>Level 2a	Students reported improved knowledge on opioid use and management. Medical students had a statistically significant lowest reported scores when compared to all other professions.<br><br>Medical students were the least likely to agree with statements reflecting positive attitudes toward IPE, consistently scoring lowest on items such as "working with students enhances my education" and "participating in educational experiences with other professions improves my future ability to work in an interdisciplinary team."
**[59]**	USA	Post-intervention	41 first year medical students	50-minute interactive, pharmacist-led case-based session on migraine pharmacotherapy. Students	Migraine pharmacotherapy (pharmacolo	Objective: Five NBME questions for end-of-block summative	End of second year	Level 1<br><br>Level 2b	Students rated the session highly (4.4–4.5/5.0).

				reviewed drug charts pre-class and worked through a 12-question clinical case in pairs during class. Pharmacist-led discussion followed each question.	gy knowledge)	examinations<br><br>Subjective:<br>Five likert-style questions to evaluate pharmacist	 <br>End of 8-week block		Statistically significant improvement on NBME questions related to session content compared to national average (90.6% vs 82.6%, p=0.0052).
**[53]**	USA	Post-intervention	21 fourth-year medical students and 5 pharmacy students	Medical students, pharmacy students and at least one staff from each faculty went into Zoom breakout rooms. Medical students presented patient cases and consulted pharmacy students. Group discussions regarding medical misinformation, interprofessional collaboration and scarce resource allocation conducted by faculty members.	Interprofessional collaboration in COVID-19 case management (interprofessional collaboration)	Subjective: post-session rating on a 5-point scale	Immediately after	Level 1	Rated the quality of the session as “Very Good” (4/5) and rated the instruction as “Excellent” (4.6/5)
**[56]**	Australia	Post-intervention	386 fifth year medical students (60%)<br>254 third year pharmacy students (40%)	Intervention consisted of three phases: Asynchronous, synchronous and post workshop reflection. Intervention created for students to practice interprofessional communication using a simulated EMR software (EHRgo).	Patient admission and collaborative medication plan using EMR (Prescribing skills)	Subjective:<br>Evaluation of learning activity on a 5-point Likert-scale (relevance to practice, achievement of learning outcomes, organisation and quality)	Immediately after	Level 1<br><br>Level 1	94% of students agreed or strongly agreed activity was relevant, 84% achieved learning outcomes, 74% felt it was organised well and 79% found it to be a quality learning activity.<br><br>Written feedback highlighted three key themes: students valued the meaningful interprofessional discussions, praised the

				All 640 students participated in a synchronous Zoom workshop facilitated by clinical educators from pharmacy and medicine. The session centred on reviewing pre-workshop materials and collaboratively developing a medication plan for a simulated patient.		Written feedback on intervention			design of the activity, but noted that facilitators were not sufficiently engaged or supportive during the session.
**[48]**	UK	Post-intervention	20 third year medical students and 14 fourth year pharmacy students	Two-hour interprofessional case-based workshop consisting of medical and pharmacy students. Focus on establishing an accurate drug history, providing information about medicines, detect and documenting adverse drug reactions and writing appropriate prescriptions. The workshop was facilitated medical and pharmacy professionals.	Prescribing skills (Prescribing skills)	Subjective: Semi-structured interviews exploring session recall, knowledge/skill retention, application to practice, and attitudes to interprofessional collaboration.	1 – 2 years post-IPE session	Level 1<br>Level 2a<br>Level 2a	Participants enjoyed meeting and interacting with other health professionals<br>Medical students recognised pharmacy students' knowledge in therapeutics and prescribing<br>Medical students gained an understanding of the role of pharmacists
**Didactic learning**									
**[64]**	Australia	Cluster randomised controlled trial	4 of 8 interns and 3 of 8 residents attended in Feb; 4 of 8 interns in Apr (total of 11 doctors-in-training)	I: Surgical interns, residents, and clinical pharmacists attended a single 30-minute in-person group education session led by the hospital's analgesic stewardship	Educating doctors-in-training and pharmacists to reduce prescription	Objective: Proportion of patients prescribed slow-release opioids at discharge	Baseline period (1 February 2018 – 30 April 2018)	Level 4b	15% reduction of slow-release opioids in intervention group (p<0.001)<br>6.4% reduction in control (p=0.001).<br>Adjusted incident rate ratio 0.52 (48% less slow-release opioids prescribed in intervention group compared to control)

			All clinical pharmacists attended (8 in Feb, 6 in Apr)<br><br>Opioid naïve patients	pharmacist.<br><br>C: Junior medical officers and pharmacy staff received routine continuing education. However, this did not include the content from the intervention sessions.	of opioids (Prescribing skills)	Secondary outcomes: proportion discharged without opioids; dose and quantity of opioids; inclusion of de-escalation plans.	Post-intervention period (17 February 2019 – 30 April 2019)		
**[34]**	India	Pre- and post-intervention	151 resident doctors	Two-hour educational session conducted by pharmacovigilance coordinator, deputy coordinator and head of department of pharmacology. Session 1 (30 mins) introductory lecture. Session 2 (60mins) slide presentations covering adverse drug reactions (ADR). Session 3 (20 min) practical demonstration on ADR form filling. Session 4 (10mins) Post training questionnaires filled out	Pharmacovigilance knowledge, attitudes, and ADR reporting practice (pharmacology knowledge)	Objective: KAP questionnaire focusing on knowledge on ADR reporting and pharmacovigilance programme of India<br><br>Subjective: KAP questionnaire focusing on doctors attitudes	Immediately before and after	Level 1<br><br>Level 2b	93.3% found the session useful and relevant and 90% rated content and teaching methods as excellent. Participants appreciated hands-on Vigiflow component.<br><br>Mean score for knowledge on pharmacovigilance and ADR improved from 8.91±2.11 to 12.90±2.04 ($p<0.05$) after intervention.
**[28]**	Saudi Arabia	Pre-post intervention	114 fourth-year female medical students	Pharmacist-led education module covering six online formal lectures via zoom, prescription writing in conjunction with e-prescribing software, and structured hospital-based pharmacy practice (divided into three	Pharmacological knowledge and prescribing skills (Prescribing skills)	Objective: 20-item knowledge test divided into 3 domains<br><br>Subjective: Post-course satisfaction ratings (Likert scale)	Immediately before and after	Level 1<br><br>Level 2b	73% rated the experience as “Excellent”, 15% as “Very good”, 11% as “good” and 1% as fair.<br><br>Knowledge scores significantly improved from pre- to post-intervention (mean score 9.51 → 16.04; $p<0.001$). All 3 domains (pharmacy practice, pharmacy innovation

				domains pharmacy practice, pharmacy innovation, and clinical pharmacy)		and open-ended feedback			and clinical pharmacy) showed statistically significant gains (p<0.001).
**[30]**	USA	Pre-post intervention	97 learners participated, 64 completed surveys: Medicine (69%), Pharmacy (17%), Physician Assistant Studies (13%), Nursing (1%); 56% students, 41% residents/fellows	Brief opioid prescribing educational intervention delivered during a month-long geriatric rotation. Included a live-streamed didactic focused on geriatric-specific opioid risks, screening for opioid use disorder, and harm reduction strategies. Didactic sessions delivered over zoom.	Opioid prescribing in older adults (Pharmacology knowledge)	Subjective: Learner-reported knowledge and confidence using 5-point Likert scale	Immediately before and after	Level 1<br><br>Level 1	High learner satisfaction with teaching clarity (mean=4.72/5), perceived clinical enhancement (4.75/5), overall rotation satisfaction (4.81/5); 91% agreed the session would help provide better patient care and educate patients.<br><br>Significant increase in learner-reported knowledge (2.81 → 3.80, p=0.0001) and confidence in suggesting non-opioid treatments
**[38]**	Canada	Pre- and post-intervention	13 surgical trainees	A 2-day pharmacist-led interactive prescribing curriculum for surgical residents focused on surgical prescribing errors, safe documentation, and antibiotic use.	Prescribing errors (Prescribing skills)	Objective**:** Number of prescription errors	Immediately before and monthly for 6 months after intervention	Level 3	A statistically significant reduction in monthly medication prescribing errors was observed three months after the intervention (p=0.035), along with a significant decrease in prescription writing errors at the same time point (p=0.046). However, no significant changes were noted in medical prescribing errors four months post-intervention, and there were no significant differences in decision-making errors related to prescribing.
**Simulation and role-play**									

**[52]**	New Zealand	Post-intervention	Three medical and three pharmacy students fourth-year students	Real-time cloud-based interprofessional simulation (SimPHARM). Pairs of pharmacy and medical students managed a virtual patient case over two days, followed by a debrief session and questionnaire.	Therapeutic decision-making; interprofessional interaction (interprofessional collaboration)	Subjective: Purpose-built post-intervention 14 item questionnaire<br><br>SimPHARM-log analysis of comments left by students and interviews	Immediately after	Level 1<br><br>Level 2a	"High" (5/5) ratings for ease of use, ability of access and ability to perform what students wanted (e.g. order lab tests, leave notes)<br><br>Medical students reported "moderate" (equivalent to 3/5) from the learning and interactions with their pharmacist partners. Moderate for this simulation improving students understanding as a future doctor. Pharmacy students rated this as high.
**[39]**	USA	Pre-post intervention	16 medical, 20 pharmacy, 40 nursing students and 2 physician assistant students substituting as medical students	Five four-hour multistation interprofessional simulation focusing on medication error prevention and management. Teams completed four case-based scenarios with embedded medication errors. Each scenario included simulation, debrief, and analysis of "huddle" behaviours. Preceded by TeamSTEPPS® Essentials training.	Medication error prevention (Prescribing skills)	Objective: Video-coded huddle behaviour (frequency, size, tightness); performance of scenario-specific critical behaviours<br><br>Subjective: TeamSTEPPS® Teamwork Attitudes Questionnaire (T-TAQ) before and after	Immediately before and after	Level 2a<br><br>Level 4b	Significant improvement in teamwork attitudes across nearly all T-TAQ subdomains: structure, leader, situation monitoring and communication. No improvement in mutual support subdomain.<br><br>Objective video data showed larger huddles, increased participation, and longer huddle duration over time. Greater huddle time was inversely associated with patient harm and care delays ($p<0.003$).
**[61]**	South Korea	Pre-post intervention with control	41 medical, 46 nursing, and 29 pharmacy final year students	One-day IPE program (6 hours) involving small-group activities and role-play based on a medication error scenario.	Interprofessional collaboration and	Subjective: 17 questions measuring perceptions towards IPE (PIPE), 15 items	Immediately before and after	Level 1	Improvements in self-efficacy scores ($p=0.011$). Most students satisfied with the program (Average >4/5) but unsatisfactory

			I: 21 medical, 23 nursing, and 14 pharmacy students<br><br>C: 20 medical, 23 nursing, and 15 pharmacy students.	Roles and expectations of doctors, nurses and pharmacists also discussed. Developed collaboratively by faculty from three disciplines medicine, nursing and pharmacy.	medication error (interprofessional collaboration)	examining self-efficacy for interprofessional experiential learning and 9 questions examining perception towards interprofessional competency<br><br>Separate 15 item questionnaire to examine satisfaction with education		Level 2a	for the duration of the program (average 3.71/5) being too short.<br><br>Significant improvement in PIPE scores post intervention (p=0.001). Interprofessional competency scores improved significantly in all domains (collaborative leadership, communication skills, interprofessional conflict solving skills, understanding the roles of other professionals and understanding their own roles within the collaborate practice) between intervention and control group.
**[32]**	Australia	Pre- and post-intervention	8 final-year medical students. 8 final-year pharmacy students, 8 final-year nursing students	A six-hour interprofessional, in-person workshop covered topics such as pre-screening and consent, vaccination area setup, safe handling of sharps and needlestick injury prevention, managing adverse reactions and anaphylaxis, and practicing intramuscular and subcutaneous injection techniques. The training and assessment were jointly conducted by a nurse and a pharmacist.	Vaccination skills and interprofessional teamwork (pharmacology knowledge)	Subjective:<br>19 item readiness for Interprofessional Learning Scale (RIPLS) with 5-point Likert items	Immediately before and after	Level 1<br><br>Level 2a	Statistically significant improvement in perceived knowledge, perceived knowledge and confidence with influenza vaccination training (p<0.05)<br><br>No significant change in medical students' attitudes to interprofessional administration of vaccines

**[40]**	Germany	Pre-post intervention	224 fifth-year medical students attended; 56 conducted a simulated prescription talk	Simulation-based pharmacology module on prescription communication. Students conducted a simulated prescription talk with a trained actor (first spontaneous, then repeated after guided peer feedback). Feedback was moderated by a pharmacist using a structured conversation guide.	Doctor–patient communication in prescribing; pharmacology education (Prescribing skills)	Objective: Content analysis of simulated prescription talks before and after peer feedback; Written test assessing prescription communication 4 days later	Immediately before and after; written test at 4 days	Level 2b	Significant improvement in comprehensiveness of prescription talks after guided peer discussion: adverse effects (2 → 37/38 students), consent (14 → 29), progress evaluation (15 → 31). In the follow-up test, students who participated (as "doctor" or observer) outperformed non-participants across most categories of communication content. Active participants performed better than observers.
**[29]**	USA	Pre-post intervention	137 medical students and 145 pharmacy students	Interprofessional telehealth medication error disclosure simulation involving pharmacy and medical students over zoom. Included pre-simulation training (podcast, patient case review), simulated telehealth encounter to disclose medication errors, and structured interprofessional faculty-led debrief.	Medication reconciliation (pharmacology knowledge)	Subjective: 9 item survey using 5-point Likert scale	Immediately before and after	Level 1<br><br>Level 2a	Significant improvement in all students' self-reported confidence in medication error disclosure (p<0.001) and comfort using telehealth technology (p<0.001).<br><br>Significant improvement in understanding of community pharmacist's role in medication therapy management (p<0.05).
**[42]**	USA	Pre- and post-intervention	69 medical students (22.7%)<br>111 pharmacy students (36.5%)<br>92 nursing students (30.3%)<br>14 athletic training students (4.6%)	A 110-minute live Zoom session was conducted, with 4–5 students per breakout room. During the session, students interviewed a standardized patient and developed a management plan, followed by	Pharmacotherapy for alcohol and opioid misuse (pharmacology knowledge)	Objective: Survey to evaluate knowledge (8 MCQ)<br><br>Subjective: Survey to evaluate confidence (6 statements)	Immediately before and after	Level 1<br><br>Level 2b	Confidence: Medical students had a significant improvement in confidence (p<0.001)<br><br>Knowledge: Medical students had a statistically significant improvement post intervention (p<0.001)

			7 social work students (2.3%)<br>5 addiction studies students (1.6%)<br>3 prevention science students (1%)<br>3 nursing master's students (1%)	a facilitator-led debrief and peer feedback.<br>The interprofessional activity was designed by a collaborative team from nursing, medicine, pharmacy, and social work across two U.S. universities.				
**[41]**	USA	Pre-post intervention	20 family medicine residents<br>14 responded to survey (1 fourth-year medical student, 4 PGY1, 4 PGY2 and 5 PGY3)	One-hour didactic lecture and one-hour hands-on simulation lab co-led by a physician, registered nurse, pharmacist, and pharmacy resident. Covered continuous glucose monitors (CGMs), diabetes medications (GLP-1, SGLT2, insulin), safety, and patient access resources.	Diabetes medication management and interprofessional prescribing education (pharmacology knowledge)	Objective: 7-item diabetes knowledge test (multiple choice + select all that apply).<br><br>Subjective: 5-item confidence survey (Likert scale).	Immediately before and after	Level 1<br><br>Level 2b	All learners (n=13) improved their confidence score by at least 1 point.<br><br>Mean knowledge scores improved from 57% to 70% ($p < .05$), though 27% of learners scored lower post-training.
**[37]**	USA	Pre- and post-intervention	298 second year medical students	A 60-minute in-person interprofessional workshop was held over two days, during which pairs of fourth-year pharmacy students facilitated and guided groups of ten second-year medical students. Workshop content developed by one physician, two pharmacists and a fourth-year pharmacy student. Workshop	Prescription writing and e-prescribing skills (Prescribing skills)	Objective: 11-question knowledge test, 30-minute e-prescribing case<br><br>Subjective: 11-item confidence survey with a 5-point Likert-scale	Immediately before and after	Level 1<br><br>Level 2b	Statistically significant improvement in confidence and satisfaction with assessment ($p<0.001$).<br><br>Statistically significant improvement in 9/11 questions on knowledge test (all 9 had $p<0.001$). Knowledge improved from 52% to 79% ($p<0.001$). 7% of students correctly completed e-prescribing assessment prior, improved to 51% post intervention.

				included writing practice, weight-based dosing, a simulated e-prescribing activity, and patient counselling techniques.					
**[62]**	Germany	Pre- and post-intervention with control	154 third-year medical students and 67 nursing trainees<br><br>IG (66 medical students and 28 nursing trainees)<br>CG (88 medical students and 39 nursing trainees)	C: A 60-minute uniprofessional simulation involving six medical students focused on communication skills related to medication errors, followed by a 60-minute session where students observed other interviewers.<br><br>I: A 90-minute interprofessional simulation involving six medical students and three nursing students focused on developing communication skills related to medication errors.<br><br>An interprofessional communication skills simulation was designed by a collaborative team comprising health services researchers, psychologists, and registered nurses.	Interprofessional communication surrounding medication errors (Prescribing skills)	Subjective: German Interprofessional Attitudes Scale (G-IPAS) and five-point Likert scale student evaluation of 'Interprofessional Communication Error' scale<br><br>Semi-structured interviews for formative evaluation	Immediately before and after<br><br>Immediately after	Level 1<br><br>Level 2a	The organisation of the activity was viewed positively; however, there was an imbalance in professional representation, with a higher number of medical students. As a result, nursing students were required to take on multiple roles. Additionally, participants faced challenges in delivering interprofessional feedback on errors.<br><br>There was a statistically significant improvement in the 'Interprofessional Communication Error' scale (p < 0.001) and in the 'Teamwork, Roles, and Responsibilities' subscale (p = 0.012). However, scores on the 'Patient-Centeredness' subscale were similar between groups and remained unchanged following the intervention.<br><br>.
**Work-integrated-learning**									

**[46]**	UK	Pre-post intervention	27 first year and 28 second year doctors-in-training	Emails written by a pharmacist containing prescribing tips and advice were sent to doctors-in-training. Contents of emails were based off ward pharmacist identified prescribing errors.	Prescribing skills	Objective: Number of prescribing errors	Immediately before and after	Level 4b	Statistically significant reduction (p<0.05) in prescribing error rate between pre-intervention (7.1%) and post intervention (5%). Both medical and surgical wards reported a significant reduction in prescribing error rates.
**[26]**	USA	Pre-post intervention	204 medical and 116 pharmacy students	Full-day interprofessional home visit program “No Place Like Home” (NPLH) where teams of 1 medical + 1 pharmacy student conducted 4–6 home visits under supervision of a geriatrics-trained clinician. Focus on interprofessional collaboration, shared decision-making, and communication.	Interprofessional collaboration in home-based clinical care (interprofessional collaboration)	Objective: Preceptor assessments via modified Team Objective Structured Clinical Encounter (TOSCE) 4-point scale<br><br>Subjective: ICCAS-R retrospective pre-post survey (20 items)	After the intervention (retrospective self-assessment)	Level 2a	ICCAS-R showed significant improvement in all 20 interprofessional competency domains (p<.001); 79% reported their ability to collaborate with other professions was “Somewhat” or “Much Better”
**[60]**	USA	Pre-post intervention with control	36 paediatric residents (10 intervention, 26 control)	Two-week pharmacist-delivered pharmacotherapy rotation (PTR) in the PICU. Residents joined the pharmacy team, participated in structured pharmacotherapy discussions, acted as clinical pharmacist during rounds, and completed daily debriefs with the pharmacist preceptor.	Prescribing skills and pharmacological knowledge (pharmacology knowledge)	Objective: 19-item multiple choice knowledge test<br><br>Subjective: 9-item confidence scale (Likert)<br>ISVS (Interprofessional Socialization and Valuing Scale -	Immediately before and after	Level 2a<br><br>Level 2b	Significant pre-post gains on ISVS total score (p=0.004) and in all subscale’s ability (p=0.006), value (p=0.005), and comfort (p=0.005). Residents reported enhanced appreciation for pharmacists' roles and interprofessional teamwork.<br><br>Pharmacotherapy knowledge improved from 12.86 → 14.29 (p=0.02); confidence from 4.70 → 5.65 (p=0.00009) for intervention

				Control group included all other residents in PICU rotation.		intervention group only)			group. Control group had no significant changes.
**[49]**	Poland	Post-intervention	6 final year medical and 7 final year pharmacy students	Groups of 2-3 medical/pharmacy students conducted telehealth consultations with patients focusing on medication reviews. Consultations were followed by mentor-guided discussions with an experienced physician and pharmacist who provided feedback on student recommendations for patients.	Geriatric medication management via telemedicine (Prescribing skills)	Subjective:<br>In-depth interview with 2 medical (male and female) and 2 pharmacy (male and female) pharmacy students	4-8 weeks after	Level 1<br><br>Level 2a	Students expressed satisfaction with the knowledge, skills, and attitudes they gained through the project.<br><br>Students enhanced their trust and appreciation of the other profession.
**[50]**	Netherlands	Post-intervention	6 Bachelors of Medicine, 18 master of medicine, 4 pharmacy, 4 physician assistant and 2 advanced nursing students<br><br>32 patients reviewed; patients were aged 70 or older, suspected of cognitive decline and used more than 5 chronic medications	Interprofessional student-led medication review program (ISP) during outpatient geriatric clinic visits. Teams of 3 students conducted full medication reviews (history, review, case discussion, feedback, implementation, follow-up). Supervised by clinical pharmacologist and multidisciplinary staff.	Interprofessional collaboration on polypharmacy (interprofessional collaboration)	Objective: Number and type of medication-related problems identified; number of medication changes advised and implemented<br><br>Subjective: Digital student survey consisting of 20 multi-choice and	Immediately after	Level 1<br><br>Level 2a	Students rated each step of the medication review process to be educational (median 4-5/5) and rated the program a median 85/100.<br><br>Students reported improved understanding of other professions' expertise and limitations; 91% valued interprofessional work. Students reported that the program would stimulate further interprofessional teamwork (median 4/5)

						open-ended questions		Level 4b	Students identified 14 medication-related problems and proposed 95 changes, of which 68 (71.6%) were implemented.
**[16]**	USA	Retrospective pre-post intervention	32 residents<br><br>53 patients with chronic opioid prescription reviewed	2-3 residents, 2-3 pharmacy students, physician faculty member and pharmacist faculty member reviewed several patients focusing on opioid prescription. Recommendations were given to each patient's primary care physician.	Opioid prescribing practice in chronic pain management (Prescribing skills)	Objective: Change in morphine milligram equivalents (MME); adherence to written recommendations (n=514)<br><br>Subjective: Resident interviews on perceived learning and satisfaction	Immediately before and 6 months post-intervention	Level 1<br><br>Level 4b	All seven residents interviewed reported peer-review intervention was effective use of time and indicated a desire to continue it.<br><br>Significant reduction in average morphine milligram equivalent from 147.75 to 133.05 (p=0.035). Total number of recommendations followed and proportion of recommendations followed were correlated with a decrease in MME (p=0.004 and = 0.013, respectively)
**[57]**	Scotland	Post-intervention	10 GP trainees and 9 pharmacists	Pharmacists were trained to participate in a mandatory prescribing workplace-based assessment (WPBA) for final-year GP trainees. Pharmacists provided feedback on prescribing alongside GP trainers. Sessions lasted for 2.5 hours.	Prescribing safety, interprofessional collaboration (interprofessional collaboration)	Subjective: Focus group interviews using prepared topic guide to discuss advantages and disadvantages of pharmacist involvement	Immediately after	Level 2a	Four main themes emerged from focus group interviews: Improved understanding and relationships between pharmacists and final year GP trainees, improved prescribing safety, interprofessional collaborative learning and considerations of pharmacists in the future. GP trainees valued pharmacist feedback, noted different perspectives on prescribing safety, and reported gaining confidence in interprofessional discussions.

**[51]**	South Africa	Post-intervention	Unspecified number of students. Approx 24-32 medical and pharmacy students split evenly. Medical students ranged from 3rd – 6th years, pharmacy students were 3rd years.	Students volunteered at a student-run clinic (Trinity Health Services) providing primary care to the homeless. Involved paired consultation, peer collaboration, supervised decision-making, and interprofessional role-sharing.	Primary health care, social accountability, interprofessional collaboration (interprofessional collaboration)	Subjective: Focus group discussions transcribed and thematically analysed	Immediately after	Level 1<br><br>Level 2a	Students described the clinic as a safe, engaging, and meaningful learning environment.<br><br>Reported improvements in communication, confidence, interprofessional role clarity, and patient-centred care. Identified value in learning from both supervisors and peers. Medical students recognise pharmacy students' expertise in pharmacology knowledge.
**[27]**	USA	Pre-post intervention	9 PGY1/PGY2 family medicine residents and 9 fourth-year pharmacy students	Pharmacy students and family medicine residents were paired during a 3–4-week inpatient rotation to provide patient care. Involved daily collaboration, shared care planning, and weekly didactic/presentation components.	Interprofessional collaboration in inpatient care (interprofessional collaboration)	Subjective: SPICE-2 survey (10 items on IPC perceptions<br><br>Optional narrative comments	Immediately before and after<br><br>Immediately after	Level 2a<br><br>Level 2a	Family resident doctors had a statistical improvement in 2/10 SPICE-2 items relating to understanding of pharmacy training requirements and a reduction in healthcare costs with IPC.<br><br>FM residents reported pharmacists provided great value to patient care, improvement in medication selection and felt more comfortable interacting with pharmacists.

**[36]**	USA	Pre-post intervention	47 internal medicine residents surveyed (11 responses)<br><br>395 inpatients	Interprofessional team of eight internal medicine residents, one internal medicine faculty and four pharmacists met with groups of residents every 5 weeks. Intervention aimed to educate internal medicine residents on inpatient hyperglycaemia management using audits and feedback. Consisted of 3 'plan-study-do-act' cycles.	Inpatient hyperglycaemia management (Prescribing skills)	Objective: Proportion of glucose values > 180mg/dL in non-critical care resident ward patients<br><br>Subjective: Survey based off Kirkpatrick model	Pre (June–July 2022) and post (Aug 2022–Mar 2023)	Level 1<br><br>Level 4b	81% of residents agreed that the intervention was helpful and would recommend it. 90% agreed that their management of hyperglycaemia had improved because of the feedback.<br><br>Statistically significant reduction of 25% of glucose values > 180mg/dL to 23% (p<0.05).
**Multicomponent learning intervention**									
**[43]**	USA	Pre- and post-intervention	67 first year medical students and 97 third-year pharmacy students	Teams of 1-2 pharmacy students paired with 1 medicine student. Three-hour IP session on pharmacogenomics. Three components: 1. Pharmacogenomics-focused patient case discussion 2. Prescription writing and review 3. Genotyping of students (CYP2C19*2) (case-based, experiential)	Pharmacogenomics and prescription writing (pharmacology knowledge) and (Prescribing skills)	Subjective:<br>9 item questionnaire on confidence and in pharmacogenomics knowledge and perceptions of interprofessional roles	Before and after IP experience	Level 1<br><br>Level 2a	Improved confidence in using pharmacogenomics in patient cases<br><br>No statistically significant result in response to "The pharmacist does not play a role in pharmacogenetic data interpretation and application" – medical students strongly disagreed before and after intervention

**[63]**	New Zealand	Pre-post intervention with control	C: 31 PGY1 residents across two health services<br><br>I: 10 PGY1 residents across two health services	Adapted ePPiFany prescribing education program: pharmacist coaching, two video-recorded simulations with feedback, and structured interprofessional ward support over 3 months. (didactic, simulation, work-integrated-learning)	Prescribing safety, pharmacist-physician collaboration (Prescribing skills)	Objective: Prescribing error prevalence by resident doctors<br><br>Subjective: Semi-structured interviews	Over a 6-month period divided into two 3-month blocks (control, intervention). Interviews immediately after	Level 1<br><br>Level 4b	Rated positively by residents, they valued this intervention early in their training<br><br>Error prevalence dropped significantly: Site 1 by 79% (p=0.02); severity of major/severe errors also reduced to zero. Site 2 showed smaller, non-significant improvements (p=0.35)
**[33]**	Vietnam	Pre-post intervention	Physicians working in two internal medicine study wards<br><br>298 geriatric patients (152 pre, 148 post)	Pharmacist-initiated educational intervention: 2-hour training sessions for physicians on medication reconciliation, audit-feedback, SOP introduction, and 2-week pharmacist support (retroactive MedRec and discussion of unintentional medication discrepancies (UMD) at 24h post-admission).<br>(didactic and work-integrated learning)	Medication reconciliation at hospital admission (Prescribing skills)	Objective: Proportion of patients with at least one UMD at 24h after admission<br><br>Proportion of patients with at least a preventable ADE score ≥0.1 due to an UMD.	Immediately before and after	Level 4b	Significant reduction in patients with at least one UMD from 55.3% to 25.3% (ORadj 0.255 95% CI: 0.151 – 0.431, p < 0.001). Significant reduction in proportion of patients with preventable ADE score ≥0.1 from 44.1% to 11.6% (OR 0.188, 95% CI: 0.105-0.340, p < 0.001).

**[54]**	USA	Post-intervention	300 third-year medical students and 100 third-year pharmacy students<br><br>Only 236 responses to survey were included	Half-day synchronous educational program via Zoom divided into four activity-based sessions. 1. Listened to a family member tell a story of about medication error, 2. Students collaborated to map prescription processes, 3. post-discharge medication reconciliation role play 4. Error disclosure simulation (didactic, team based, role play and simulation)	Interprofessional collaboration for patient safety (Prescribing skills)	Subjective:<br>24 item questionnaire: 17 questions rated on a 5-point Likert scale and 7 qualitative questions	Immediately after	Level 1<br><br>Level 2a	87.9% of medical students responded favourably to interprofessional collaboration<br><br>"It made me re-assess how important my role is as a physician, and how important it is to work closely with the pharmacist and make sure the patient it is getting the medications he/she needs"
**[44]**	Indonesia	Pre- and post-intervention	78 fourth-year medical students and 58 first-year pharmacy students	Simulation on prescription services for 3h, for 3 days. Prescription services involve paediatric case-based scenarios requiring students to prescribe and dispense medication.<br><br>Home visits (8h) for 2 days which were guided by a doctor and a pharmacist. Medical and pharmacy students create a management plan for each patient and present to facilitators. (simulation and work- integrated-learning)	Prescription services (paediatrics) and home visits (Prescribing skills)	Subjective:<br>20 item Inter-professional Collaborative Competency Attainment Scale (ICCAS) questionnaire	Immediately before and after	Level 2a	There was a statistically significant improvement ($p < 0.05$) across all six domains: communication, collaboration, understanding of roles and responsibilities, a collaborative patient/family-centred approach, conflict management and resolution, and overall team functioning.

**[58]**	USA	Post-intervention	26 resident physicians and 8 pharmacy residents	1–2-hour activity involving two 15-minute presentations on pulmonary function tests, COPD and asthma diagnosis; overview of various types of inhalers. Presentations were followed by hands on group activity to manipulate inhalers in conjunction with a case activity. (didactic and case based)	Inhaler technique and promoting interprofessional collaboration (interprofessional collaboration)	Subjective: Open-ended survey questions and interviews exploring perceptions of interprofessional roles, practice changes, and perceived barriers.	Immediately after	Level 2a	Four themes emerged from survey questions: 1. Improved understanding of other professions' contributions, (2) Belief that patient care improves with interprofessional input, (3) Recognition of personal knowledge gaps, and (4) Time and coordination as barriers.
**[55]**	UK	Post-intervention	9 fifth (final) year medical students and 9 3rd/4th year pharmacy students	Students participated in three structured online interprofessional education (IPE) sessions focused on hospital discharge planning. Each session involved collaborative completion of a discharge summary, simulated patient counselling, and reflection. Students were assessed using an adapted discharge summary rubric. (case-based learning and simulation)	Interprofessional collaboration during discharge planning (interprofessional collaboration)	Objective: Structured assessment of discharge summaries (15-point rubric across 3 domains: completeness, quality, presentation) Subjective: Student feedback on usefulness, realism, and teamwork	Immediately after	Level 1<br><br>Level 2b	92% of students rated the sessions as useful; students highlighted improved confidence, realism, and value of cross-professional feedback. All students agreed the discharge summary exercise was an effective IPE tool.<br><br>Mean discharge summary scores improved significantly across the three sessions ($p = 0.01$), indicating increased competence in team-based documentation. No significant difference between professions ($p = 0.681$).

**[45]**	Germany	Pre and post-intervention	59 medical students and 46 pharmacy students over three semesters	Students first watched two videos on multimorbidity and medication safety in preparation. Medical and pharmacy students were paired together to conduct a medication review with case examples. Students then participated in a patient consultation role play with feedback from a doctor and a pharmacist. Students also tandem shadowed at a primary care centre and discussed about potential drug-related issues with the teaching physician. (case based and work-integrated-learning)	Interprofessional collaboration in outpatient care; polypharmacy and medication safety (interprofessional collaboration)	Subjective:<br>5-point Liker scale and open-ended questions<br><br>SPICE-2D questionnaire (Student perceptions of Physician-Pharmacist Interprofessional clinical education)	Immediately before and after	Level 1<br><br>Level 2a	Of the 46 respondents, 45 would recommend the in-person interprofessional learning activity and 37 were satisfied with their learning process<br><br>SPICE-2D scores increased across all three semesters for medical students. No statistical testing reported.
**[35]**	Canada	Pre- and post-intervention	13 family medicine PGY3 residents	Pharmacist-led one-month foundational lecture to medical residents focusing on promoting collaborative learning and HIV pharmacotherapy. Learning tasks included a mixture of case-based learning and real patient consults.<br>(didactic, case based and work-integrated-learning)	HIV pharmacotherapy (pharmacology knowledge)	Subjective:<br>Survey to evaluate confidence in knowledge on 5-point Likert-scale<br><br>Written feedback on educational intervention<br><br>Behavioural changes	Immediately before and after	Level 1<br><br>Level 1<br><br>Level 3	No statistical analysis was conducted; however, survey results indicated an increase in confidence, though the extent of this improvement is difficult to quantify.<br><br>Written feedback from students strongly supported the intervention.<br><br>Some students were observed taking comprehensive medication histories, utilising HIV-specific pharmacotherapy

									resources, and focusing on identifying drug-related problems.
**[11]**	Netherlands	Retrospective pre-post intervention	274 medical students and 35 pharmacy students	IPE program consisted of three separate activities: 1. students address a case and discuss (1st year of masters) 2. Students engage in discussions focusing on pharmacogenetics (2nd year of masters) 3. Students conduct a medication review for a polypharmacy patient in primary practice (2nd year of masters). All activities had an uniprofessional and an interprofessional component. medical students experienced both IPE and UPE; pharmacy students participated only in IPE (case based and work-integrated-learning)	Pharmacotherapy; interprofessional collaboration (pharmacology knowledge)	Subjective: 20-item self-report tool – Interprofessional Collaborative Competency Attainment Scale (ICCAS) and subject interviews	Participants received ICCAS over a ten-month period	Level 2a	All 20 ICCAS demonstrated statistically significant improvements with both UPE and IPE ($p<0.05$). IPE had greater effect sizes compared to UPE with 1 item in collaboration and 1 item in roles and responsibilities having large effect sizes (Cohen's $d > 0.8$). 6 items for IPE had medium effect sizes, all UPE effect sizes were small. Subject interviews highlighted student reported improvements valuing each professions expertise, understanding different roles, improving communication with one another and improving teamwork (sharing leadership).
